# Common founder effects of hereditary hemochromatosis, Wilson´s disease, the long QT syndrome and autosomal recessive deafness caused by two novel mutations in the *WHRN* and *TMC1* genes

**DOI:** 10.1186/s41065-017-0052-2

**Published:** 2017-12-19

**Authors:** K. Sigvard Olsson, Olof Wålinder, Ulf Jansson, Maria Wilbe, Marie-Louise Bondeson, Eva-Lena Stattin, Ruma Raha-Chowdhury, Roger Williams

**Affiliations:** 1 Section of Hematology and Coagulation, Department of Medicine, Sahlgrenska Academy, University of Göteborg, S 413 45 Göteborg, Sweden; 2 Department of Medicine, Östersund Hospital, Östersund, Sweden; 3 Department of Clinical Chemistry, Sundsvall Hospital, Sundsvall, Sweden; 4 Department of Immunology, Genetics and Pathology, Science for Life Laboratory, Uppsala University, Uppsala, Sweden; 5 Department of Immunology, Genetics and Pathology, Uppsala University, Uppsala, Sweden; 6 John van Geest Centre for Brain Repair, Department of Clinical Neurosciences, University of Cambridge, Cambridge, UK; 7 Institute of Hepatology London, Foundation for Liver Research, London,SE5 9NT and Faculty of Life Sciences & Medicine, King´s College London, London, UK

**Keywords:** Hereditary hemochromatosis, Wilson´s disease, Long QT syndrome, Jervell and Lange- Nielsen´s syndrome, Non syndromic hearing loss, *WHRN*, *DFNB31*, *TMC1*

## Abstract

**Background:**

Genealogy and molecular genetic studies of a Swedish river valley population resulted in a large pedigree, showing that the hereditary hemochromatosis (HH) *HFE/p.*C282Y mutation is inherited with other recessive disorders such as Wilson´s disease (WND), a rare recessive disorder of copper overload. The population also contain individuals with the Swedish long QT syndrome (LQTS1) founder mutation (*KCNQ1*/p.Y111C) which in homozygotes causes the Jervell & Lange Nielsen syndrome (JLNS) and hearing loss (HL).

Aims of the study were to test whether the Swedish long QT founder mutation originated in an ancestral HFE family and if carriers had an increased risk for hemochromatosis (HH), a treatable disorder. We also aimed to identify the pathogenic mutation causing the hearing loss disorder segregating in the pedigree.

**Methods:**

LQTS patients were asked about their ancestry and possible origin in a HH family. They were also offered a predictive testing for the HFE genotype. Church books were screened for families with hearing loss. One HH family had two members with hearing loss, who underwent molecular genetic analysis of the LQTS founder mutation, connexin 26 and thereafter exome sequencing. Another family with hearing loss in repeat generations was also analyzed for connexin 26 and underwent exome sequencing.

**Results:**

Of nine LQTS patients studied, four carried a HFE mutation (two p.C282Y, two p.H63D), none was homozygous. Three LQTS patients confirmed origin in a female founder ( b 1694, identical to AJ b 1694, a HFE pedigree member from the Fax river. Her descent of 44 HH families, included also 29 families with hearing loss (HL) suggesting JLNS. Eleven LQTS probands confirmed origin in a second founder couple (b 1614/1605) in which the woman b 1605 was identical to a HFE pedigree member from the Fjällsjö river. In her descent there were not only 64 HH, six WND families, one JLNS, but also 48 hearing loss families. Most hearing loss was non syndromic and caused by founder effects of the late 16^th^ century. One was of Swedish origin carrying the *WHRN,* c.1977delC, (p.S660Afs*30) mutation, the other was a *TMC1*(NM_138691),c.1814T>C,(p.L605P) mutation, possibly of Finnish origin.

**Conclusions:**

Deep human HFE genealogies show HFE to be associated with other genetic disorders like Wilson´s disease, LQTS, JLNS, and autosomal recessive hearing loss. Two new homozygous HL mutations in *WHRN*/p.S660Afs*30 and *TMC1/*p.L605P were identified,none of them previously reported from Scandinavia. The rarity of JLNS was possibly caused by miscarriage or intrauterine death. Most hearing loss (81.7%) was seen after 1844 when first cousin marriages were permitted. However, only 10 (10.3%) came from 1^st^ cousin unions and only 2 (2.0 %) was born out of wedlock.

**Electronic supplementary material:**

The online version of this article (10.1186/s41065-017-0052-2) contains supplementary material, which is available to authorized users.

## Background

River valley populations of northern Sweden are well suited for research of monogenic diseases because the wilderness between them creates sub-isolates [1]. This usually mean a good breeding ground for autosomal recessive (AR) diseases like hereditary hemochromatosis (HH), common in the river valleys of Jämtland [2, 3]. In this disease, homozygous carriers of the *HFE*/p.C282Y mutation have an increased ability to absorb iron due to reduced levels of hepcidin, the iron regulating hormone of the liver [4].With time, the iron loading can reach toxic amounts affecting multiple organs in particular the liver, but most often the phenotype is mild. The disease was once regarded rare in Scandinavia and England, possibly due to widespread iron deficiencies in these countries [5]. Paradoxically these and other north European countries today demonstrate the highest mutation frequencies in the world, suggesting selective advantages [6, 7]. A recent study seemed to indicate that that the *HFE*/p.C282Y could segregate other mutations, when Wilson´s disease (WND), a rare recessive copper loading disorder [8, 9] appeared in a HH family of the Swedish west coast. Shortly afterwards this was repeated in a deep HFE family of the Fjällsjö river in northern Jämtland [10]. The Swedish long QT syndrome (LQTS) founder mutation is now reported to originate in this population [11].

LQTS is an autosomal dominant inherited cardiac arrhythmic disorder which in homozygotes also causes hearing loss, and the Jervell and Lange- Nielsen syndrome (JLNS) [12], a surprisingly rare disorder, according to a recent study by the same authors [13]. By extensive genealogic investigations the authors were able to trace individuals carrying the long QT mutation (*KCNQ1/*p.Y111C) to common founders, one, a female born 1694 [14], the other a couple born 1605 and 1614 [11]. The origin and geographic spread of the LQTS mutation, described in detailed maps [11] fascinated us as it seemed to coincide with the p.C282Y mutation [2] in its upstream spread into Lapland. This land of 15000 km^2^ north of N 63.9 was opened for colonization by the Lapland Bill of 1673, a matter of great relief for expanding post war populations such as the Fjällsjö (from *n* = 91 in 1620 to *n* = 299 in 1751) [15]. As an upstream migration would most likely result in matches between mutation carriers and risk for homozygous children [2, 3, 16] we would expect not only HH but also JLNS along the lines of descent. Despite a law change in 1844 permitting 1^st^ cousin unions [17], JLNS was surprisingly rare as only one (p.Y111C/p.Y111C) afflicted individual have been found [13]. Singular JLNS subjects have however, been reported [18] and according to a parish meeting protocol (from 1831) there were families with deaf mute children in the new habitat [19]. Assuming they may represent historic JLNS, church registries were screened for hearing loss families.

The only way at this time to identify the two LQTS founder families [11, 14] was to ask systematically LQTS patients about their ancestry and its possible origin in hemochromatosis founder families. Like HH patients [2, 3] LQTS families have been detected and reported [20] from the county hospital in Östersund and subsequently by other workers (Olof Lövheim and Anders Gard) at the same hospital.

### Aims

The purpose of this study was to find out whether patients with the Swedish LQTS founder mutation (*KCNQ1*/p.Y111C) had an increased risk for HH, by sharing origin within hemochromatosis (HH) families. We also aimed to determine the origin and the molecular genetic background of the hearing loss disorder segregating in the pedigree.

## Methods

After obtaining permission from their doctors, LQTS patients from 11 families were contacted and asked about their ancestry. Before contact they were given written information about hemochromatosis and the aims of the study. Hearing loss patients were also contacted and all consented to an interview, and gave written consent to blood tests. The study was approved by the Regional Ethical Review Board at the University of Göteborg, (*Dnr 834/14) ,T 214/16 and T1076-16). G*enealogic data were extended through information from the genealogists Mr Georg Hansson and Mr Olof Stenum as previously described [2, 10] and the results were registered in our hemochromatosis/ Wilson disease database, Holger 7, (http://5493.shop.textalk.se) comprising n = 11076 individuals [10]. Added to the same registry was the ancestry of families with deaf or deaf- mute children detected in church registries by Mr Stenum. During the interviewing process additional hemochromatosis patients were identified and added to the registry that now comprises (*n* = 13720) individuals. Results were given in pedigrees drawn manually in Cyrillic 2.1 (Cyrillic Software, Old Beaconsfield, UK). In hemochromatosis patients serum iron, transferrin saturation (TS), serum ferritin, liver enzymes and HFE mutations were analyzed as previously described [2]. LQTS patients were tested for HFE mutations in the LightCycler™ (Roche Diagnostics Gmbh Mannheim, Germany). Sequencing of the *KCNQ1/*p.Y111C LQTS founder mutation was performed at the laboratory of Clinical Genetics, Umeå University hospital [14], Umeå, Sweden. Sequencing and multiplex ligation probe amplification (MLPA) of the *GJB2*(Cx26) gene was performed at department of Clinical Genetics, Uppsala University, Uppsala, Sweden. Whole exome sequencing was performed by using Ion AmpliSeq™ Exome Library preparation protocol (Thermo Fisher and sequencing by the Ion Chef system as described in Bondeson et al [21]. Primer sequences and PCR conditions for Sanger sequencing of *WHRN* (NM_001173425), c.1977delC, (p.S660Afs*30), and *TMC1*(NM_138691), c.T1814C,(p.L605P) are available upon request.

### Setting

The study area included the Angerman river and its tributaries the Fax river and the Fjällsjö river [2, 11]. In 1974 the study area was divided with the upstream parishes Fjällsjö, Bodum and Tåsjö being transferred to the county of Jämtland. This might have been of importance because the single county hospital in Östersund happened to dispose of a laboratory equipment (Autochemist) including routine determinations of serum iron and transferrin saturation (TS) [2, 3]. As an elevated TS is an early biochemical marker of iron overload [4], this gave us unique opportunities to detect hemochromatosis.

## Results

### Three hypotheses were examined

1. The LQTS founders originated in HFE families

1 A. Is the first *KCNQ1*/Y111C founder, a woman b 1694 [14] identical to A J b 1694? If so she is seen as V:16 in our Fax river hemochromatosis pedigree, Additional file 1: Figure S1 [2].

1 B. Is the second founder family b 1614 and 1605 [11] identical to KA b 1614 married to I O b 1605? If so they are identical to III:1 and III:2 in our Fjällsjö river hemochromatosis pedigree , (Additional file 1: Figure S2) [2].

2. If true, there would be a high probability for JLNS in the descent of 1 A, AJ b 1694 and 1 B, IO b 1605, and

3. An increased risk for hemochromatosis in LQTS carriers.

**1 A. The first** ***KCNQ1*** **/Y111C founder, a woman b 1694** [13] **is identical to AJ b 1694, a hemochromatosis pedigree member of the Fax river** [2].

**Fig. 1:**
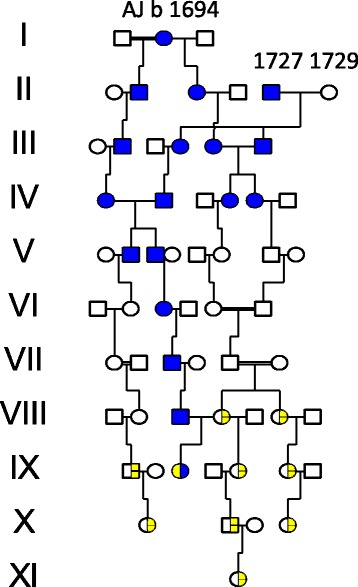
Pedigree showing individuals with Long QT Syndrome (LQTS) and hereditary hemochromatosis (HH). LQTS are marked with half filled symbols, hemochromatosis with filled blue symbols. Double horisontal lines mark consanguineous marriages

Three LQTS families described an ancestry as shown in Fig. 1 in agreement with the pedigree described by Winbo et al in their first study [14]. They shared origin in AJ b 1694, identical to V:16 in our Fax river hemochromatosis pedigree [2] (see Additional file 1: Figure S1). AJ had moved in from the Fax river (see family 526 www.5560.se/familjer/fam-str.pdf to settle new land in Lapland upstream in the Fjällsjö river valley in 1732. Her two children (belonging to different marriages) had a descent who married descent members of another pioneer couple b 1727/1729, their closest neighbor living 4 km downstream the Fjällsjö river. AJ:s daughter II:3 b 1739 died at age 32 from smallpox but had with her husband 6 children who survived to reproduce.

**1 B. The second** ***KCNQ1*** **/Y111C founder family b 1614 and 1605** [11] **is identical to KA b 1614 married to I O b 1605, a hemochromatosis family member of the Fjällsjö river** [2]**.**

**Fig. 2:**
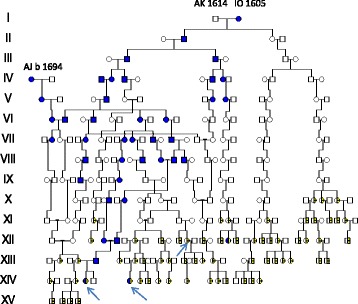
Pedigree connecting families with LQTS and HH to a common founder couple b 1614/1605. IV:1 is identical to AJ b 1694 from Fig. 1 and V:3-V4 b 1729 /1727 were her neighbors . Double heterozygotes (LQT/HFE) are marked by lower arrows. XII:12 ,marked by a lower left arrow, had a hearing aid at age 42. Symbols as in Fig. 1

**Fig. 3:**
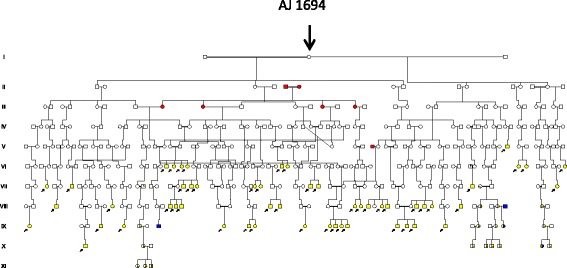
Pedigree showing individuals with hearing loss marked with filled yellow symbols with a short lower arrow (suspected Jervell & Lange-Nielsen Syndrome). LQTS as in Fig. 1. Admixture from an outside origin is marked with filled red symbols

Eleven long QT families including the three of Fig. 1 revealed that they all originated in a founder family born 1614/1605 identical to III:1 and III:2 of our Fjällsjö river HFE-pedigree (Additional file 1: Figure S2) [2]. These findings shown in Fig. 2 agree well with the Winbo et al pedigree /Fig. 3/ [11] supporting our second hypothesis. The first LQTS founder AJ b 1694 is seen as IV:1 and the 1727/1729 family of Fig. 1 now appears as V:3-4 and is identical to V:5 in the Winbo et al pedigree /Fig. 3/ [11], which connected 10 (38.4%) of the 26 long QT families. Repeated consanguineous marriages were seen, however without hearing loss other than in XII:12. The degree of coinheritance with *HFE*/p.C282Y was substantial but involved only the upstream migration while the downstream descent from II:3 seemed unaffected by the p.C282Y.

**2 A. Hearing loss suggesting JLNS in the descent of a LQTS founder b 1694** [14]**.**

The descent of the first founder AJ b 1694 comprised 25 A4 pages in agreement with a high reproduction and a high frequency of HH families (*n* = 49). For clarity these were excluded in the pedigree of Fig. 3, which contained not only four 4 LQTS families, but also 29 families with deaf mute children suggesting JLNS. Phenotypic expression other than hearing loss was most often mild and sudden death was rarely reported.

**2 B. Hearing loss suggesting JLNS in the descent of the LQTS 1605/1614 founder family.**

**Fig. 4:**
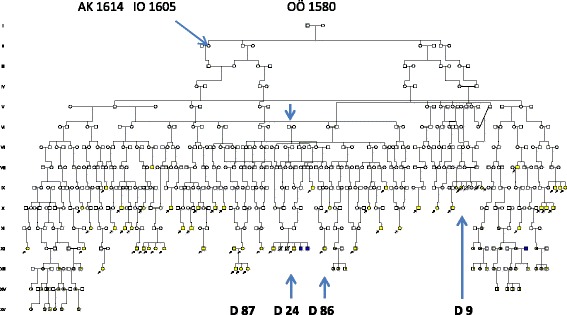
Pedigree showing families with LQTS and hearing loss (suspected Jervell and Lange Nielsen syndrome (JLNS). **D 9** was deaf-mute and died of stroke at age 9 suggesting JLNS. The pedigree also show connection to a hemochromatosis founder family born 1580 in which three HL families **D 87**, **D 24** and **D 86** were available for molecular genetic evaluation. The **D 24** parents were 3^rd^ cousins once removed from a marriage 1788 marked with a vertical arrow. The LQTS founder II family is marked by a left upper arrow. Symbols as in Fig. 2

The descent of the second founder family comprised 106 A4 pages and was difficult to illustrate in Cyrillic, mainly due to the high number of HH families (*n* = 64), of whom 35 (55%) were in common with the first founder AJ. There were also six WND families [10] and 48 families with hearing loss of whom 15 were in common with the first founder. A simplified pedigree is seen in Fig. 4 including a 1^st^ cousin marriage (III:1-III:2) and the descent from one (IV:1) of their 11 children. In her descent was the **D 9** family with a nine year old daughter who died on the 4^th^ of October 1839 ‘af slag, döfstum’ = (from stroke, deaf- mute). This was shortly after her mother´s death, aged 33, during her 5^th^ childbirth on 14^th^ of September 1839. As emotional stress may initiate serious arrhythmic events, this might have been the background of the “stroke”. The mother had lost another stillbirth and also three other children, of whom two died within their first three days of life. The parents of the 9 year old girl were only distantly related to the LQTS 1605/1614 founder II family.

The **D 24 family** was of interest because of the two deaf mute siblings XII:18 and XII:21were still alive in 2014 and available for a molecular genetic evaluation. Their two brothers with HH , XII:22 and XII:23 had been previously reported as homozygous carriers of the HLA A3B7 haplotype [2], (See: S2 XIII:43-44, down right arrow) suggesting a common origin. In support of a possible JLNS was the sudden death of their brother XII:19 at age 14 (stroke of lightning) and a record from 1967 when the oldest brother XII:18, was observed at hospital after fainting. An ECG showed however, normal QT duration and a subsequent test negative for the *KCNQ1*/p.Y111C mutation excluded the possibility of JLNS.

Mutation analysis of the *GJB2* gene encoding connexin 26, did not reveal any mutations in families D 24, D 86 and D 87 thus excluding DFNB1, the most common form of autosomal recessive HL. Exome sequencing of XII:18 and XII: 21 **(**Fig. 4) following a recessive filtering towards an in-house database (consisting of 1400 ethically matched controls) and publicly available database revealed an interesting finding .One novel homozygous variant, c.1977delC,(p.S660Afs*30) in the *WHRN* gene (also denoted *DFNB31*) (NM_001173425),was found which*,* encodes the whirlin protein. Mutations in *WHRN* have previously been shown to cause autosomal recessive non syndromic deafness [22] and Usher syndrome type 2D [23]. The novel variant c.1977delC,(p.S660Afs*30**)** was considererd to be pathogenic since it results in a frame-shift, and is present in homozygous form in the family and the normal population frequency is very low. Segregation analysis of the p.S660Afs*30 was performed in **D 86** and in XII:16 of the **D 87** family, and none of the individuals investigated carried the mutation.

In the **D87** family exome sequencing was performed of the two unrelated HL parents XII:16 and XII:15 (from a distant river valley), and the youngest of their three deaf mute children, XIII:12, following a recessive filtering as described above. All family members were homozygous for a novel missense variant in the *TMC1* gene c.T1814C,(p.L605P), (NM_138691). Mutations in the *TMC1* gene are causing autosomal recessive HL [24] and is one of the most commonly mutated genes in the Western-European population [25]. The variant c.1814C,(p.L605P) was considered to be likely pathogenic since it is present in extremely low frequency in the normal population and is located in the TMC domain. There are multiple lines of computational evidence that support a deleterious effect and it is present in homozygous form in the family. The *TMC1* mutation was not identified in **D 24** and **D 86** supporting the presence of at least two different HL mutations in the population. The hearing loss in **D 86,** was most probably caused by his mother´s rubeola infection.

### Origin of mutations

**Fig. 5:**
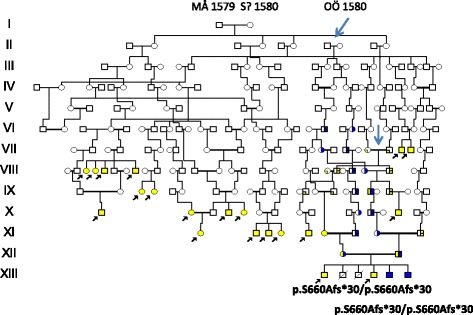
Pedigree of the **D 24** family presenting the origin and segregation of the p.S660Afs*30 mutation of the *WHRN DFNB31*gene. Two siblings from a marriage in 1778 (arrow) may have introduced the mutation by co migration with the HFE mutation (in blue). A founder effect of the late 16^th^ century seems likely. VI:1-2 is equal to II:5-6 of Fig. 3

Parents of the **D 24** family with the *WHRN/p.*S660Afs*30 mutation were 3^rd^ cousins once removed from a remote consanguineous marriage in 1778 marked by an arrow in Fig. 4. Further extension revealed a possible founder origin in a family of the late 16^th^ century from village K in the parish of Anundsjö. This founder family seen in Fig. 5 had a descent with similarly affected HL families living in the parish. The VI:1-2 family (family size 14, effective family size 6) had members who migrated into the new habitat. They are recognized in the pedigree of Fig. 3 (marked in red). The village K family (comprising 139 A4 pages) had a total of 60 HL families in its descent (data not shown), but the **D 87** was not included suggesting a different origin of that mutation.

**Fig. 6:**
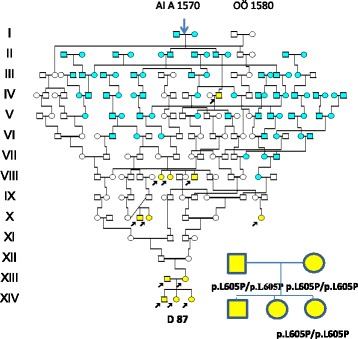
Pedigree showing the ancestry of the **D 87** family with homozygosity for the *TMC1/p.*L605P in repeat generations. Both parents of XIII:2 share origin in a generation VIII family with hearing loss. The first HL member observed (on the 23^rd^ of December 1715 (44) is seen as IV:16. IV:18 is the “Finnkommissioner” of the HFE family marrying into the Finnish founder family. Finnish ancestry is marked with light blue symbols

**Fig. 7:**
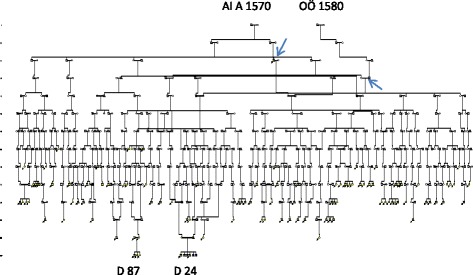
A pedigree connecting 68 hearing loss families including the **D 87** family with the *TMC1*/p.L605P mutation to the common founder of Fig. 6. The first documented hearing loss individual (MP b 1655), is marked by an upper arrow and the “Finncommisioner” with a lower arrow

The mother of the **D 87** family, homozygous for the *TMC1/*L605P mutation, originated from a consanguineous marriage in which both parents shared origin in a HL family of the late 18^th^ century as seen in Fig. 6. Further extension revealed a common origin in a Finnish migrant family of the late 16^th^ century. Their descent is seen in Fig. 7 connecting 68 HL families (of whom 54 were in common with the village K family). Although extensive, the pedigree is incomplete because for clarity, nine WND families, seven LQTS and 75 HFE families had to be excluded. Most hearing loss families (81.4%) were seen after 1844 when first cousin marriages were permitted. However, only 10 (10,3 %) came from 1^st^ cousin unions, there were 11 (11.7%) of 2^nd^ cousins, 16 (17.0 %) 3^rd^ and 5 (5.3 %) 4^th^ cousins. A remote consanguinity was present in 18(19.1 %). Only 2 (3.6 %) were born out of wedlock.

**3. LQTS patients carrying HFE mutations.**

Nine LQTS patients were available for a HFE genotype test, two carried p.C282Y, two p.H63D and five were without HFE mutations. None was homozygous.

Electrocardiograms have not been rechecked in all of HH probands, only in those with a history of arrhythmia. None was found to have prolonged QT duration. One retested was a 43 year old male in the descent of LQTS founder II family who had serious arrhythmic events needing implantable cardioverter defibrillator (ICD) treatment. He had normal hearing and normal QT intervals but had a severe HH phenotype (to be published).

## Discussion

In the present study we could verify the first and second hypotheses but not the third. The genealogic studies suggest that the Swedish LQTS founder mutation *KCNQ1*/p.Y111C [11, 14] shared an origin and segregated with the HFE mutation [2] in a river valley population of northern Sweden (Figs 1, 2, and 3 and Additional file 1: Figure S1 and Figure S2) **.** It is known that genetic material with potentially modifying properties may co-segregate with the deleterious mutation [11], however, there is little evidence to support iron or HFE mutations [26, 27], as contributing factors to the mild phenotype seen in the Swedish LQTS families [11, 14]. The map findings of LQTS upstream the river [11] are fascinating, not only for corresponding with HFE, but also for the hearing loss families along the same pathways. Such families existed in the new habitat because a parish meeting protocol (of 1831) informed that “deaf mute children at age 11 to 14 years had a chance of a free place at a hearing institute” [19]. There would be a high probability for *KCNQ1*/p.Y111C homozygosity and JLNS [13], because of a preference of LQTS mutation carriers to marry within the same river valley [1, 11, 14] and also because first cousin unions were no longer prohibited by law. This law change in 1844 had been shown to have a major influence on hereditary diseases in northern Sweden [17]. The consanguineous marriages shown in Fig. 1 and the findings of 29 hearing loss families shown in Fig. 3, was therefore not surprising. They could well represent JLNS, but against such a diagnosis was a phenotype in which sudden death was rarely reported. Similar observations were made in the descent of the second LQTS founder (Fig. 4) with one exception, the **D 9** family, which was severely affected and may well represent JLNS. Three other individuals afflicted with JLNS from the same hospital [18] may belong to this founder family of Fig. 4. It is possible that homozygous pregnancies were selected against resulting in early abortion or intrauterine death of their outcome [13]. As we have found such families to occur, and also families with multiple miscarriages, this may explain the rarity of JLNS. Surprising was the rarity of JLNS in inbred LQTS families with repeat consanguineous marriages seen in Fig. 2. However, homozygous carriers without hearing loss have been known since 1998 [28] and may contain a 10% residual *KCNQ1* function that rescues hearing [29]. In such high-risk families fetal heart rate observations have been helpful to predict the phenotype in LQTS [30].

### Origin and nature of non syndromic hearing loss detected by exome sequencing

The genetic tests of the **D 24** family excluded the JLNS and DFNB1 because screening of the LQTS *KCNQ1/*p.Y111C and *GJB2* did not reveal homozygous or compound heterozygous mutations [31]. However , exome sequencing revealed the presence of a mutation *WHRN/p.*S660Afs*30 at the DFNB31 locus [22].This mutation was possibly introduced in a marriage of 1778 seen as VI:15-16 of Fig. 4 (marked by an arrow). Further genealogic studies supported the presence of a local founder effect with hearing loss families connected through consanguineous marriages in the parish of Anundsjö as shown in Fig. 5. There was a considerable spread of the supposed *WHRN* gene from the founder family into the new habitat and the VI:1-2 of Fig. 5 is an example with a significant effect (marked in red) upon the pedigree of Fig. 3. The DFNB31 locus was first identified in a Palestinian family living in Jordan [22] and in a Tunisian kindred [32], but is not a common cause of hearing loss [22].

We were surprised that the **D87** family, with hearing loss in successive generations [18], lacked the *WHRN/*S660Afs*30 mutation. However, exome sequencing of the two HL parents and one of their three HL children showed all three to be homozygous for *TMC1/*p.L605P. The *TMC1* gene has previously been reported to cause autosomal recessive hearing loss [24]. Like *WHRN*, it is also reported from Tunisia [33] and Morocco [34], but also from Western [25] and Eastern [35] Europe. Extensive genealogic studies seen in Figs. 6 and 7 now support a possible Finnish founder origin. This vast pedigree and the pedigree of Fig. 5 may well explain most of the hearing loss of previous pedigrees, Figs. 3 and 4.

We can not exclude the possibility of single cases being caused by environmental factors such as infections, (rubeola and cytomegalovirus) . Nor can we exclude HL cases for being misclassified as feeble-minded in the church records. Previous reports of hearing loss in Sweden showed the highest figure, (13/10000) in the county of Västerbotten [36], almost identical to the figures from Finland , the highest in Europe [37]. More recent studies (81) support the high Västerbotten figures and the HL findings of Fig. 7 may be seen as a background.

Somewhat remarkable were the present findings of a deep HFE genealogy [2] upstream a Swedish river valley segregating with Wilson´s disease [10] and LQTS [11, 14] was also segregating with hearing loss caused by mutations in *WHRN* and *TMC1* respectively*.*

The HFE mutation has, despite its young age, reached surprisingly high frequencies in North European iron poor areas [2–4, 6, 7] as predicted by Motulsky [38]. The lack of hepcidin giving homozygotes an increased capacity to absorb iron [4] might have been of selective advantage in an iron poor environment like the present Swedish ones [2, 39]. Whether the immune response genes in their HLA has contributed is hard to prove, but the haplotypes, A1B8 of the Fax river and A3B7 of the Fjällsjö river [2] are both very common, showing a striking relationship with HFE/p.C282Y through Europe [6].

A possible explanation to a selective advantages of *HFE* , *LQTS* and *TMC1* is their presence in early colonizers (Figs. 1-4, 6-7 ) of the new Lapland habitat. As compared to individuals in the wake, the pioneers at a colonization front are said to be much more successful in passing their genes on to future generations through a mechanism of gene surfing [40]. Whether such a factor might have influenced the spread of the *WHRN* mutation seen in Fig. 5 is unclear. The family VI:1-2 of this pedigree consisted of 14 siblings of whom six (all females) reproduced, equal to an effective family size (EFS) of six. Three stayed and married in the local parish and got children with an average EFS of two (4+1+1), while three migrated into the new habitat and married colonizers (marked in red of Fig. 3). They got an average EFS of 7.7 (6+8+9) suggesting selective advantages. However, their success might also have been influenced by their marriage partners of past Finnish origin, from the first colonizers of Lapland well described by Richard Gothe [41]. Extensive genealogic studies seen in Figs. 6 and 7, suggest a possible Finnish origin of the *TMC1* mutation. The founder AI A was born about 1570 in Äänekoski, a parish of central Finland. Our findings may be questioned because *TMC1* has not been found among 6612 Finnish alleles tested in the Exome Aggregation (ExAC) database [42] in which 5 *TMC1* alleles were found from Europe. However, our findings may be supported by the Finnish deaf mute statistics of 1912 (revised by Björkquist 1916 [43]) in which the parish of Äänekoski reported 11 (corresponding to 19/10000) HL individuals.

The first document of hearing loss in the new habitat is from an inspection of the church in Arsilla = Åsele in 1715 [44]. On the 23^rd^ of December 1715 the inspector arrives in Hällan, the first village in Lapland at which he had to spend the night in the house of a colonizer who was “dumbe”= deaf mute. The next day he passes the village Gafsele and the house of the “Finncommissioner” until arrival in Åsele. There is genealogic evidence that the colonizer b 1655 is identical to IV:16 of Fig. 6 and the “Finncommissioner” identical to IV:18. The two are marked with arrows in Fig. 7.

Selective advantages was recently demonstrated in a similar upstream isolate of the Saguenay river, (SLSJ), of Quebec, Canada, in which deep human genealogies revealed a selective advantage to those on an expanding wave front [45]. The genetic footprints of these pioneers are still recognizable, and the HLA-A3B7 haplotype of the **D 24** HFE family, also present in (SLSJ) [46], was recently attracted attention because of its presence in an Irish skeleton of the bronze age [47].

It may seem strange that a founder common to one disease (HH) is a common founder also of another disease. However this is not uncommon according to lessons from Quebec [48]. The findings of Hereditary hemochromatosis [2], Wilsons disease [10], LQTS [11, 14], and Hereditary hearing loss in the present study is therefore not unique.

Our pedigrees are small in number compared to the great French Canadian (SLSJ) findings [48], however this may be explained by the harder environmental conditions in Sweden north of N 63.9. While the upstream SLSJ today have a population of about 300.000 [48] less than 15000 live in the Swedish habitat (*n* = 12396, Statistics Sweden 2015).

One simple explanation for the survival and multiplication of the HFE mutation is its often mild phenotypic expression in homozygotes [49]. Even in those with a severe phenotype this comes late and does not interfere with reproduction. Two previous studies have been unable to prove reproductive advantage in hemochromatosis families [50, 51]. Whether the same would be seen in an iron poor environment as the present [2–4, 39], is unknown.

Somewhat surprising was the low frequency (22.2%) of HFE alleles (two p. C282Y, two p.H63D) in LQTS carriers. None was homozygous or showed compound heterozygosity.

Finally, recent studies using exome sequencing have shown that individuals can carry multiple recessive mutations as demonstrated in the Hutterites population [52]. In their study, some individuals could carry up to 5 recessive mutations and some could be homozygous for 3 different mutations . Our findings of two new recessive hearing loss mutations segregating with HFE [2], WND [10] and LQTS [11, 14] is therefore not remarkable, and is possible to reveal due to new technology now available.

Even if genealogic studies may be fascinating they are also time consuming. We would therefore recommend the use of information already assembled in genealogic registries. It is likely that some of the remaining 30% of the LQTS probands could have been attached [11]. Even if we agree that all blood lines are not conceived within wedlock [11], recent studies using Y-chromosome and genealogical data, have shown low historical rates of cuckoldry [53].

## Conclusions

Deep human HFE genealogies show HFE to be associated with other genetic disorders like Wilson´s disease, LQTS, JLNS, and autosomal recessive hearing loss. Two new homozygous HL mutations in *WHRN*/p.S660Afs*30 and *TMC1/*p.L605P were identified,none of them previously reported from Scandinavia. The rarity of JLNS was possibly caused by miscarriage or intrauterine death. Most hearing loss (81.7%) was seen after 1844 when first cousin marriages were permitted. However, only 10 (10.3%) came from 1^st^ cousin unions and only 2 (2.0 %) was born out of wedlock.

## Additional file

Additional file 1: Figure S1. From Eur J Haemat 2008; 81: 36-46 Fig. 3, with permission. A female AJ b 1694? who married twice is marked by an upper right arrow. **Figure S2.** From Eur J Haemat 2008; 81: 36-46 Fig. 7, with permission. A couple AK b 1614 (III:1) and IO b 1605 (III:2) is marked by an upper left arrow. The D24 family (deafmute siblings not shown) is marked by a lower right arrow. (PDF 150 kb)

## References

[1] Einarsdottir E, Egerbladh I, Beckman L, Holmberg D, Escher SA (2007). The genetic population structure of northern Sweden and its implications for mapping genetic diseases. Hereditas.

[2] Olsson KS, Ritter B, Hansson N, Chowdhury RR (2008). HLA haplotype map of river valley populations with hemochromatosis traced through five centuries in Central Sweden. Eur J Haematol..

[3] Olsson KS, Ritter B, Raha-Chowdhury R (2010). HLA-A3-B14 and the origin of the haemochromatosis C282Y mutation: founder effects and recombination events during 12 generations in a Scandinavian family with major iron overload. Eur J Haematol.

[4] Pietrangelo A (2015). Genetics, Genetic Testing, and Management of Hemochromatosis: 15 Years Since Hepcidin. Gastroenterology..

[5] FINCH SC, FINCH CA (1955). Idiopathic hemochromatosis, an iron storage disease. A. Iron metabolism in hemochromatosis. Medicine (Baltimore).

[6] Beutler E (1), Felitti V, Gelbart T, Waalen J. Haematological effects of the C282Y HFE mutation in homozygous and heterozygous states among subjects of northern and southern European ancestry. Br J Haematol. 2003 Mar;120(5):887-893. 10.1046/j.1365-2141.2003.04215.x 12614226

[7] Distante S, Robson KJ, Graham-Campbell J, Arnaiz-Villena A, Brissot P, Worwood M (2004). The origin and spread of the HFE-C282Y haemochromatosis mutation. Hum Genet.

[8] Olsson KS, Konar J, Dufva IH, Ricksten A, Raha-Chowdhury R (2011). Was the C282Y mutation an Irish Gaelic mutation that the Vikings helped disseminate? HLA haplotype observations of hemochromatosis from the west coast of Sweden. Eur J Haematol..

[9] Olsson KS, Raha-Chowdhury R (2012). Letter to the Editor. Eur J Haematol.

[10] Olsson KS, Wålinder O, Kindmark A, Williams R (2012). Common local founder effects for Wilson's disease and hereditary hemochromatosis; mutation studies of a large family. Scand J Gastroenterol..

[11] Winbo A, Diamant UB, Rydberg A, Persson J, Jensen SM, Stattin EL. Origin of the Swedish longQT syndrome Y111C/KCNQ1 founder mutation. Heart Rhythm. 2011 Apr;8(4):541–7. Epub 2010 Nov 30 10.1016/j.hrthm.2010.11.043 21129503

[12] Jervell A, Lange-Nielsen F (1957). Congenital deaf-mutism, functional heart disease with prolongation of the Q-T interval, and sudden death. Am Heart J.

[13] Winbo A, Stattin EL, Diamant UB, Persson J, Jensen SM, Rydberg A (2012). Prevalence, mutation spectrum, and cardiac phenotype of the Jervell and Lange-Nielsen syndrome in Sweden. Europace.

[14] WinboA DUB, Stattin EL, Jensen SM, Rydberg A (2009). Low incidence of sudden cardiac death in a Swedish Y111C type 1 long-QT syndrome population. Circ Cardiovasc Genet.

[15] Palm Andersson L. The population of Sweden´s Parishes and Communes, 1571-1997. Institute of History, Göteborg University 2000. Books on Demand, Visby, Sweden.

[16] Cavalli-Sforza LL, Moroni A, Zei G (2004). Consanguinity,Inbreeding and Genetic Drift in Italy.

[17] Bittles AH, Egerbladh I (2005). The influence of past endogamy and consanguinity on genetic disorders in northern Sweden. Ann Hum Genet..

[18] Sehlin P, Holmgren G, Zakrisson J (1990). Incidence, prevalence and etiology of hearing impairment in children in the county of Västerbotten, Sweden. Scand Audiol.

[19] Nyström R. En Lappmarksbys historia. Lajksjö och dess innevånare under 17-1800-talet .AB HärjedalensTryckeri, Sveg, 1994 (Swedish) .

[20] Ritter B, Bielinski Y, Gustavsson G, Wingman H (1987). Family study of long QT syndrome in Jämtland. Hygiea.

[21] Bondeson ML, Ericson K, Gudmundsson S, Ameur A, Pontén F, Wesström J, Frykholm C, Wilbe M. A nonsense mutation in CEP55 defines a new locus for a Meckel-like syndrome, an autosomal recessive lethal fetal ciliopathy. Clin Genet. 2017 Mar 14; doi:10.1111/cge.13012. [Epub ahead of print] PubMed PMID: 28295209 10.1111/cge.13012 28295209

[22] Mburu P, Mustapha M, Varela A, Weil D, El-Amraoui A, Holme RH, Rump A, Hardisty RE, Blanchard S, Coimbra RS, Perfettini I, Parkinson N, Mallon AM, Glenister P, Rogers MJ, Paige AJ, Moir L, Clay J, Rosenthal A, Liu XZ, Blanco G, Steel KP, Petit C, Brown SD (2003). Defects in whirlin, a PDZ domain molecule involved in stereocilia elongation, cause deafness in the whirler mouse and families with DFNB31. Nat Genet..

[23] Ebermann I, Scholl HPN, Issa PC, Becirovic E, Lamprecht J, Jurklies B, Millan JM, Aller E, Mitter D, Bolz H (2007). A novel gene for Usher syndrome type 2: mutations in the long isoform of whirlin are associated with retinitis pigmentosa and sensorineural hearing loss. Hum. Genet..

[24] Kurima K, Peters LM, Yang Y, Riazuddin S, Ahmed ZM, Naz S, Arnaud D, Drury S, Mo J, Makishima T, Ghosh M, Menon PS, Deshmukh D, Oddoux C, Ostrer H, Khan S, Riazuddin S, Deininger PL, Hampton LL, Sullivan SL, Battey JF Jr, Keats BJ, Wilcox ER, Friedman TB, Griffith AJ. Dominant and recessive deafness caused by mutations of a novel gene, TMC1, required for cochlear hair-cell function. Nat Genet. 2002 Mar;30(3):277–84. Epub 2002 Feb 19 10.1038/ng842 11850618

[25] Sommen M, Schrauwen I, Vandeweyer G, Boeckx N, Corneveaux JJ, van den Ende J, Boudewyns A, De Leenheer E, Janssens S, Claes K, Verstreken M, Strenzke N, Predöhl F, Wuyts W, Mortier G, Bitner-Glindzicz M, Moser T, Coucke P, Huentelman MJ, Van Camp G (2016). DNA Diagnostics of Hereditary Hearing Loss: A Targeted Resequencing Approach Combined with a Mutation Classification System. Hum Mutat..

[26] Laudanski K, Ali H, Himmel A, Godula K, Stettmeier M, Calvocoressi L (2009). The relationship between serum ferritin levels and electrocardiogram characteristics in acutely ill patients. ExpClinCardiol..

[27] Park SK, Hu H, Wright RO, Schwartz J, Cheng Y, Sparrow D, Vokonas PS, Weisskopf MG (2009). Iron metabolism genes, low-level lead exposure, and QT interval. Environ Health Perspect.

[28] Priori SG, Schwartz PJ, Napolitano C, Bianchi L, Dennis A, De Fusco M, Brown AM, Casari G (1998). A recessive variant of the Romano-Ward long-QT syndrome?. Circulation.

[29] Bhuiyan ZA, Momenah TS, Amin AS, Al-Khadra AS, Alders M, Wilde AA, Mannens MM (2008). An intronic mutation leading to incomplete skipping of exon-2 in KCNQ1 rescues hearing in Jervell and Lange-Nielsen syndrome. Prog Biophys Mol Biol.

[30] Winbo A, Fosdal I, Lindh M, Diamant UB, Persson J, Wettrell G, Rydberg A (2015). Third Trimester Fetal Heart Rate Predicts Phenotype and Mutation Burden in theType 1 Long QT Syndrome. Circ Arrhythm Electrophysiol.

[31] Carlsson PI, Karltorp E, Carlsson-Hansén E, Åhlman H, Möller C, Vondöbeln U, Persson LA (2012). GJB2 (Connexin 26) gene mutations among hearing-impaired persons in a Swedish cohort. Acta Otolaryngol.

[32] Tlili A, Charfedine I, Lahmar I, Benzina Z, Mohamed BA, Weil D, Idriss N, Drira M, Masmoudi S, Ayadi H (2005). Identification of a novel frameshift mutation in the DFNB31/WHRN gene in a Tunisian consanguineous family with hereditary non-syndromic recessive hearing loss. Hum Mutat.

[33] Tlili A, Rebeh IB, Aifa-Hmani M, Dhouib H, Moalla J, Tlili-Chouchène J, Said MB, Lahmar I, Benzina Z, Charfedine I, Driss N, Ghorbel A, Ayadi H, Masmoudi S (2008). TMC1 but not TMC2 is responsible for autosomal recessive nonsyndromic hearing impairment in Tunisian families. Audiol Neurootol..

[34] Bakhchane A, Charoute H, Nahili H, Roky R, Rouba H, Charif M, Lenaers G, Barakat A. A novel mutation in the TMC1 gene causes non-syndromic hearing loss in a Moroccan family. Gene. 2015 Dec 10;574(1):28–33. doi:10.1016/j.gene.2015.07.075. Epub 2015 Jul 28. PubMed PMID: 26226225 10.1016/j.gene.2015.07.075 26226225

[35] Hassan MA (1), Shah AA, Szmida E, Smigiel R, Sasiadek MM, Pfister M, Blin N, Bress A. A TMC1 (transmembrane channel-like 1) mutation (p.S320R) in a Polish family with hearing impairment. J Appl Genet. 2015 Aug;56(3):311-316. 10.1007/s13353-014-0263-4 25560804

[36] Statistical Yearbook of Sweden 1920. Stockholm, Sweden: Statistic Sweden 1920.

[37] Lumio JS, Piirainen H, Paljakka P (1966). Marriages between the deaf and hereditary deafness in Finland. Acta Otolaryngol..

[38] Motulsky AG (1979). Genetics of hemochromatosis. N Engl J Med.

[39] Samuelson G, Sjölin S (1989). Nutrition and health in Swedish children 1930-1980. Three nutrition surveys in a northern Swedish county. Acta Paediatr Scand..

[40] Hallatschek O, Nelson DR (2008). Gene surfing in expanding populations. Theor Popul Biol..

[41] Gothe R. Finnkolonisationen inom Ångermanland, Södra Lappmarken och Jämtland. Saxon & Lindström 1948, Stockholm.

[42] Lek M, Karczewski KJ, Minikel EV, Samocha KE, Banks E, Fennell T (2016). Analysis of protein-coding genetic variation in 60,706 humans. Nature..

[43] Björkqvist G (1916). Om dövstumma i Finland. Dövstum-statistik för 1912. Fennia.

[44] Wallin G (1908). Resa till Åsele åhr 1715, in Wiklund K B.

[45] Moreau C, Bhérer C, Vézina H, Jomphe M, Labuda D, Excoffier L (2011). Deep human genealogies reveal a selective advantage to be on an expanding wave front. Science..

[46] de Braekeleer M, Vigneault A, Simard H (1992). Population genetics of hereditary hemochromatosis in Saguenay Lac-Saint-Jean (Quebec, Canada). Ann Genet..

[47] Cassidy LM, Martiniano R, Murphy EM, Teasdale MD, Mallory J, Hartwell B, Bradley DG (2016). Neolithic and Bronze Age migration to Ireland and establishment of the insular Atlantic genome. Proc Natl Acad Sci U S A..

[48] Scriver CR (2001). Human genetics: lessons from Quebec populations. Annu Rev Genomics Hum Genet..

[49] Asberg A, Hveem K, Krüger O, Bjerve KS (2002). Persons with screening-detected haemochromatosis: as healthy as the general population. Scand J Gastroenterol.

[50] De Braekeleer M (1993). A prevalence and fertility study of haemochromatosis in Saguenay-Lac-Saint-Jean. Ann Hum Biol..

[51] Nelson RL, Persky V, Davis F, Becker E (2001). Is hereditary hemochromatosis a balanced polymorphism: an analysis of family size among hemochromatosis heterozygotes. Hepatogastroenterology.

[52] Chong JX, Ouwenga R, Anderson RL, Waggoner DJ, Ober C (2012). A population-based study of autosomal-recessive disease-causing mutations in a founder population. Am J Hum Genet..

[53] Larmuseau MH, Vanoverbeke J, Van Geystelen A, Defraene G, Vanderheyden N, Matthys K, Wenseleers T, Decorte R (2013). Low historical rates of cuckoldry in a Western European human population traced by Y-chromosome and genealogical data. Proc Biol Sci..

