# Supplementary figures and images for: Common founder effects of hereditary hemochromatosis, Wilson´s disease, the long QT syndrome and autosomal recessive deafness caused by two novel mutations in the WHRN and TMC1 genes
Source: Hereditas. 2017 Dec 19;154:16. doi: 10.1186/s41065-017-0052-2 (PMC5735936; doi:10.1186/s41065-017-0052-2)

Supplementary figures

Supplementary Figure S1

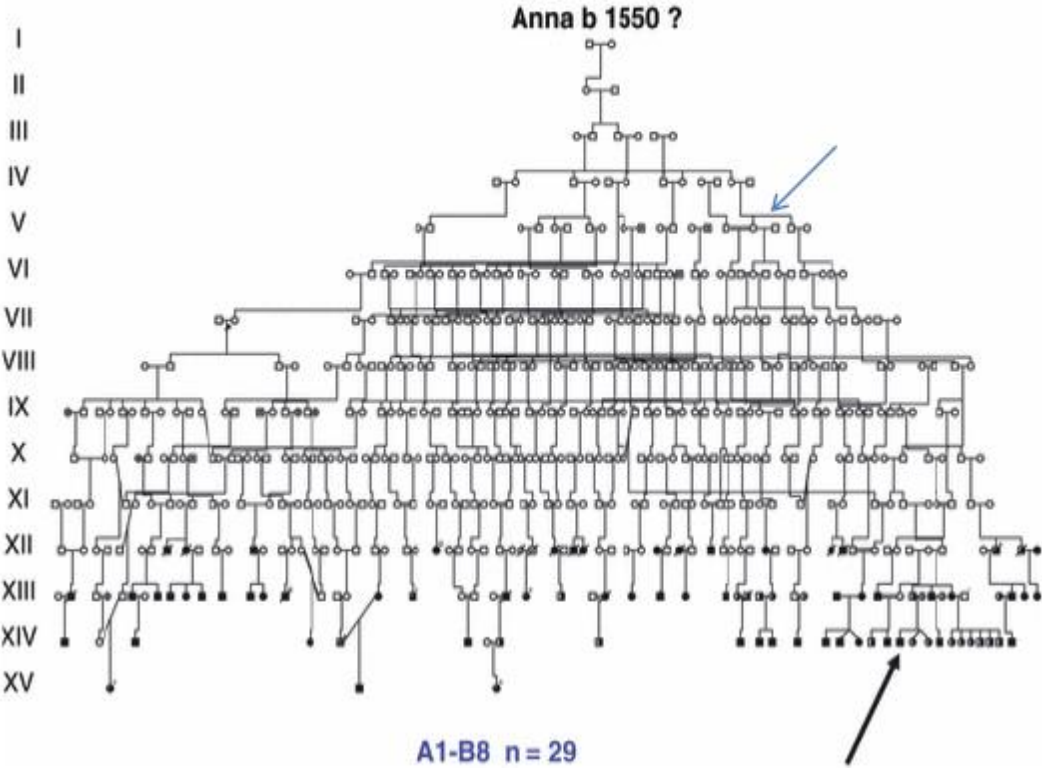

Supplementary Figure S 2

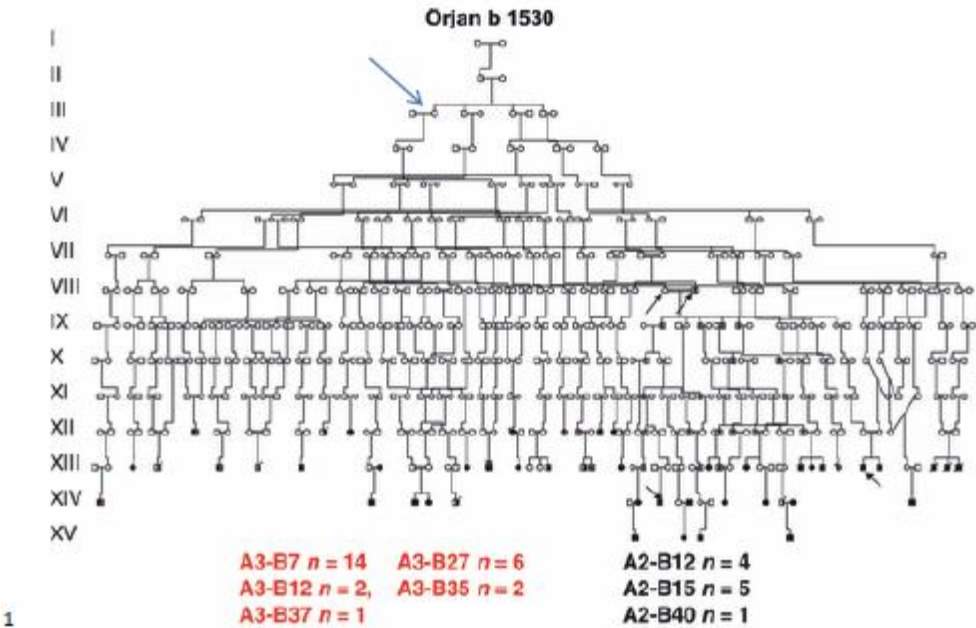

1  
2

Supplement: Additional file 1: Figure S1. — From Eur J Haemat 2008; 81: 36-46 Fig. 3, with permission. A female AJ b 1694? who married twice is marked by an upper right arrow. Figure S2. From Eur J Haemat 2008; 81: 36-46 Fig. 7, with permission. A couple AK b 1614 (III:1) and IO b 1605 (III:2) is marked by an upper left arrow. The D24 family (deafmute siblings not shown) is marked by a lower right arrow. (PDF 150 kb) [file 41065_2017_52_MOESM1_ESM.pdf]
